# The current status of radiological clinical audit - an ESR Survey of European National Radiology Societies

**DOI:** 10.1186/s13244-019-0736-4

**Published:** 2019-05-09

**Authors:** European Society of Radiology (ESR), David C Howlett, Adrian P Brady, Steve Ebdon-Jackson, Christoph D Becker

**Affiliations:** Am Gestade 1, 1010, Vienna, Austria

**Keywords:** Clinical audit, Radiology, National Radiology Society, Radiation protection, Basic Safety Standards Directive (BSSD), 2013/59/Euratom

## Abstract

The importance of clinical audit in radiological practice is increasingly recognised and undertaking clinical audit “in accordance with national procedures” is mandatory for radiology departments within the European Union following implementation of the Basic Safety Standard Directive (BSSD), 2013/59/Euratom in 2018.

This survey, sent to all National Radiological Societies in Europe in 2018, evaluated the current status of clinical audit at national level and supporting infrastructure, and explored the potential for wider co-operation and collaboration in developing and evaluating clinical audit processes.

Responses were received from 36/47 (76.6%) National Societies. Broadly responses indicated an increasing awareness of the importance of clinical audit, but highlighted deficiencies in necessary infrastructure and resources required for enhancement and development of existing clinical audit systems. National Societies are well placed, in the context of appropriate and prioritised resource allocation, to collaborate with other European bodies, in particular the European Society of Radiology (ESR), to help lead on these important changes, with the potential to provide external direction.

## Key Points

- Effective clinical audit is a key priority for all radiology departments across Europe, with the potential to improve patient care and outcomes.
- The 2018 BSSD (2013/59/Euratom) mandates undertaking radiation protection clinical audit “in accordance with national procedures” within radiology departments.
- There is increasing emphasis on external direction of internal departmental clinical audit, with oversight/accreditation provided by suitable professional, or scientific national bodies.
- 36/47 (76.6%) of European National Radiology Societies responded to a 2018 ESR survey evaluating the current status of radiological clinical audit.
- Responses revealed an appreciation of the importance and role of clinical audit, but highlighted shortfalls in necessary resourcing and infrastructure that could facilitate wider and more effective involvement of national societies.

## Introduction

Clinical audit is a well-established and valuable tool in modern healthcare systems, it is of particular relevance to radiologists due to its incorporation into the Basic Safety Standards Directive (BSSD 2013/59/Euratom) [1]. Carrying out clinical audit “in accordance with national procedures” is mandatory and a legal requirement within the European Union as a result of implementation of the updated BSSD in 2018. Clinical audit also has an important role in evaluating everyday service provision and clinical practice - regulatory clinical audit is centred around radiation protection and the requirements of the BSSD, it is a priority for all radiology departments and is compulsory.

In addition to developing processes around internal departmental clinical audit, there is a drive also to set up national processes of external audit. This might involve a multidisciplinary external auditing team working with radiology departments to carry out cross-centre external audit. Another option could include internal departmental audit with external direction. Setting up an external audit system would depend upon local/national resources and priorities, with oversight/accreditation (external direction) by a suitable professional or scientific national body, separate from the national regulatory authority (and also separate to any national processes of inspection). National radiological societies would be well placed to provide necessary oversight and co-ordination, recognising the logistic and funding implications involved.

Clinical audit is a subject of high priority for the ESR, with the ESR working collaboratively with other organisations, including the Heads of European Radiological Protection Competent Authorities (HERCA) to improve patient safety and quality of care across Europe. As part of its commitment to clinical audit the ESR produced Esperanto – the ESR Guide to Clinical Audit and Clinical Audit Tool [2] in 2017. This was widely distributed among ESR members, with copies also sent to all National Radiology Societies across Europe. A second updated version of Esperanto is to be launched at the European Congress of Radiology (ESR) in 2019 [3], with an enhanced audit section and an expanded number of 30 clinical audit templates. This development and implementation of a clinical audit tool was a key action (action 6) within the ESR EuroSafe Imaging Call For Action 2018 [4].

This paper reports the results of a survey distributed to all 47 National Radiology Societies in Europe in 2018 (a proportion of these are outside of the European Union). The survey aims:

- To establish the logistical and administrative infrastructure available for clinical audit at local and national levels.
- To evaluate awareness of Esperanto and its impact.
- To explore the challenges around developing a pan-European audit/survey network.
- To clarify national arrangements for BSSD implementation and development of responsibility for clinical audit.

This paper is best read in conjunction with the results and findings of another ESR survey undertaken at the same time, evaluating uptake of BSSD requirements (with an emphasis on clinical audit) across European EuroSafe Imaging Star radiology departments [5].

## Materials and Methods

A 14 question survey was prepared using Survey Monkey; the questions are included (with results) in Table 1. The survey also included a free text/comments section at the end. The questionnaire was distributed via the ESR office to all National Radiology Societies in Europe using a well-established network of ESR contacts with these Societies. The survey was sent out at the beginning of November 2018, for completion by the end of that month – a 2 week extension was however made available with the survey completed by mid-December 2018. The responses were received, data cleaned and results tabulated and feedback collated by the ESR office. Individual National Society responses have been kept anonymous.

**Table 1:** National Societies audit survey results

Q1	Yes - high priority	No - but becoming more important	No - not currently			
Is the promotion and development of clinical audit currently a high priority in your national society?	15 (41.67%)	17 (47.22%)	4 (11.11%)			
Q2	Yes	No	In development / under consideration			
Do you have an administrative facility within your national society responsible for the promotion and development of clinical audit?	8 (22.22%)	19 (52.78%)	9 (25.00%)			
Q3	Yes	No	Partial - some, not all			
Do you have a functional means of communication (post, email, fax) with all radiology departments in your country?	26 (72.22%)	1 (2.78%)	9 (25.00%)			
Q4	Yes	No	Yes - but not regularly			
Do you communicate on a regular basis with - and receive feedback/comments from radiology departments in your country e.g. announcement of new developments, guidelines, standards?	13 (36.11%)	4 (11.11%)	19 (52.78%)			
Q5	Yes	No	Some, not all	Don't know		
Do radiology departments in your country have adequately resourced and funded internal administrative support to support departmental participation in clinical audit?	4 (11.11%)	14 (38.89%)	15 (41.67%)	3 (8.33%)		
Q6	Central government	Local government	National society	Internal departmental resources	Industry / private finance	Other payer
If the answer to question 5 is "yes", departmental audit is funded and supported, what is the usual source of this funding (you may tick more than one response)?	5 (20.83%)	7 (29.17%)	4 (16.67%)	13 (54.17%)	3 (12.50%)	3 (12.50%)
Q7	Yes, all departments - led by national society	Partial, some departments - led by national society	Yes / partial - national society does not lead	No		
Is there a co-ordinated national programme of clinical audit in your country involving all radiology departments?	4 (11.11%)	6 (16.67%)	12 (33.33%)	14 (38.89%)		
Q8	Yes	No (skip to Q10)				
The ESR sees development and support of clinical audit as a high priority - the first edition of Esperanto, the ESR Clinical Audit Guide, was sent to national societies in Europe in 2017. Is your national society aware of this publication?	24 (66.67%)	12 (33.33%)				
Q9	Yes - sent to all	Yes - partial distribution	No - but proposed	No - and not proposed	N/A	Unaware of this publication
Has your national society distributed the ESR Esperanto Clinical Audit Guide to radiology departments in your country?	3 (12.50%)	5 (20.83%)	3 (12.50%)	9 (37.50%)	4 (16.67%)	0
Q10	Yes - mainly positive	Yes - mixed	Yes - mainly negative	No		
Has your national society had feedback from radiology departments on Esperanto?	1 (2.78%)	2 (5.56%)	0	33 (91.67%)		
Q11	Yes	Potentially	No - not at this time			
In principle would your national society support a pan European survey involving all eligible radiology departments (departmental identity kept confidential) evaluating compliance with any national regulatory requirements in place and intended to transpose Council Directive 2013/59/Euratom (BSSD) requirements?	21 (58.33%)	12 (33.33%)	3 (8.33%)			
Q12	Yes	Potentially - but internal investment needed	No - not at this time			
If a pan European survey was proposed, does your national society have the necessary infrastructure and administrative resources to support this project?	3 (8.33%)	20 (55.56%)	13 (36.11%)			
Q13	legal requirement with regulatory oversight	legal requirement with no specific oversight or checking	no specific provision at the moment			
How has the Euratom requirement that "clinical audits are carried out in accordance with national procedures" been transposed into national law in your country?	10 (27.78%)	10 (27.78%)	16 (44.44%)			
Q14	hospital-based responsibility	departmental responsibility	individual pratictioner responsibility			
How do your national procedures regulate responsibility for clinical audit?	16 (44.44%)	15 (41.67%)	5 (13.89%)			
Total rates	Response	No Response	Total			
ESR National Societies	36	11	47 (as of Nov. 2018 - 48 national societies in total)			

## Results

The results of the survey are recorded in Table 1. Responses were received from 36/47 National Radiological Societies^1^ (76.6% response rate). The results are discussed and analysed in the discussion section.

## Discussion

As previously alluded to, the development, implementation and documentation of robust processes of clinical audit is a key priority for all radiology departments across Europe. There is an appreciation of this at national level, as demonstrated in the survey, with clinical audit seen as a high priority in 42% of respondents and becoming more important in 47% (of note 11%, 4 respondents, did not see clinical audit as a high priority).

To support effective local departmental clinical audit and to provide requisite external direction at national level requires a supporting logistical and administrative infrastructure. It is clear from survey responses and individual national society feedback that there is considerable variability in the development and functionality of clinical audit systems both at local (departmental) and national society level. In some national healthcare systems audit networks are reasonably mature, resourced and functional. This is not the case however for the majority:

- 22% of national societies have an administrative facility dedicated to clinical audit.
- 72% have a functional means of communication with all radiology departments within their country.
- Only 36% are in regular communication with radiology departments.
- In only 11% of radiology departments is there a resourced and funded administrative support for audit, or a national co-ordinated programme of clinical audit.

The ESR Clinical Audit Guide, Esperanto, is a key initiative providing practical advice and support for radiology departments, particularly those who are early on in the clinical audit development pathway. The ESR has developed an enhanced version of Esperanto (for 2019) and the ESR will need to work closely with all National Radiology Societies to improve awareness of this important publication, but also to encourage wider distribution and end-user feedback on its utility at a local level.

The BSSD is legislation aimed at European Member States and how clinical audit is developed within individual member states is not specified. If an individual country devolves responsibility for clinical audit to the hospital (or employer/undertaking - see survey findings for these responses), then there would be no legal requirement for a National Society to involve itself in the undertaking of BSSD-related clinical audit. It should be noted from the survey that 16 of the respondents (44.4%) indicated there was no specific provision for transposition of the BSSD requirements around clinical audit into national law in their country at the time of the survey (these results may be subject to change, but give a 2018 baseline).

Although there might not be a legal obligation, the increased focus on clinical audit does provide an opportunity for National Radiology Societies to lead and direct national clinical audit activity. There is considerable potential for pan-European collaboration, working with European professional bodies (in particular the ESR) to develop audit programmes and to undertake multi-centre and multi-national surveys and audits. A recognition at national society level of this potential can be seen in survey responses, with 33/36 respondents in support (in reality, or potentially) of a pan-European BSSD uptake survey. The importance and potential benefits of increased national society input into local audit processes are highlighted by the findings of the companion BSSD uptake survey amongst EuroSafe Imaging Star departments [5], revealing a lack of compliance in some key BSSD radiation protection requirements. It is important to acknowledge that such developments will have resource implications, with only 3 respondents considering themselves to have the necessary infrastructure in place to support such a pan-European project.

## Conclusion

This pan-European survey of National Radiology Societies had a high response rate and is likely to be representative of European procedures and practice. The survey reveals an acknowledgement amongst National Radiology Societies of the importance of development of clinical audit processes and a desire to become more involved, working with key stakeholders like the ESR. Logistical and administrative infrastructure development is key, this will require resource prioritisation and allocation at national and local governmental level. As multi-agency co-operation and collaboration in clinical audit matures and progresses across Europe, appropriately resourced National Radiology Societies will be well placed to help lead this process.

## Footnotes

1 ESR National Radiological Societies of European countries as defined by the ESR Executive Council are listed at the following link: https://www.myESR.org/about/organisation/institutional-member-societies At the time of the survey there were 47 ESR national member societies. Subsequent to the survey a further society became ESR member. As of February 2019, the ESR counts 48 national member societies.
